# Teledentistry—Knowledge, Practice, and Attitudes of Dental Practitioners in Saudi Arabia: A Nationwide Web-Based Survey

**DOI:** 10.3390/healthcare9121682

**Published:** 2021-12-04

**Authors:** Mohammad Zakaria Nassani, Sadeq Ali Al-Maweri, Abdullah AlSheddi, Ali Alomran, Mohammed Nasser Aldawsari, Ahmed Aljubarah, Ahmed Mohammed Almuhanna, Nawaf Meshal Almutairi, Anas B. Alsalhani, Mohammed Noushad

**Affiliations:** 1 Department of Restorative and Prosthetic Dental Sciences, College of Dentistry, Dar Al Uloom University, Riyadh 13314, Saudi Arabia; inya113@yahoo.com; 2 College of Dental Medicine, QU Health, Qatar University, Doha 2713, Qatar; sadali05@hotmail.com; 3 Family Dentistry Resident, Vision College of Dentistry and Nursing, Vision Colleges, Riyadh 13226, Saudi Arabia; Dent.AlSheddi@gmail.com; 4 Cham Dental Clinic, AlKhobar 32253, Saudi Arabia; Dr.aliao95@gmail.com (A.A.); ahmedalmuhanna240@gmail.com (A.M.A.); 5 The Internship Program, Vision College of Dentistry and Nursing, Vision Colleges, Riyadh 13226, Saudi Arabia; abunasser.15@gmail.com (M.N.A.); Ahmedaljubarah2030@gmail.com (A.A.); 6 Special Security Forces Medical Centre, Dental Department, Riyadh 12321, Saudi Arabia; Nawaf59470@gmail.com; 7 Department of Oral Medicine and Diagnostic Sciences, Vision College of Dentistry and Nursing, Vision Colleges, Riyadh 13226, Saudi Arabia; Dr.anas_salhany@hotmail.com

**Keywords:** teledentistry, dental practitioners, dentists, knowledge, attitudes, Saudi Arabia

## Abstract

The present survey assessed the knowledge, attitudes, and practice of teledentistry (TD) among dental practitioners in Saudi Arabia. This questionnaire-based cross-sectional study was conducted with dental interns and practicing dentists in Saudi Arabia. An online questionnaire was sent to all potential respondents. Questions related to knowledge, practice, attitudes, and training regarding TD were presented. A total of 603 (227 dental interns, 376 practicing dentists) completed the questionnaire. Generally, the participants revealed poor knowledge and practice of TD, with only 38% having heard about TD and only one-quarter of the sample (23.2%) reporting practicing TD at their current workplace. However, most of the participants expressed positive attitudes and a willingness to practice TD in the future. Specialists and those in practice for >5 years showed significantly better knowledge and practice of TD than general dentists and those with lesser clinical experience (*p* < 0.01). While only one-fifth of the participants (20.2%) reported having attended a workshop/lecture about TD, the majority (69.7%) felt that they needed training on TD. The results revealed poor knowledge, practice, and training with regard to TD among practicing dentists in Saudi Arabia. However, the positive attitude expressed by most of the participants towards practicing TD in the future is an encouraging sign for dental educators and planners of oral health care. Continuous education through periodic workshops and training courses on TD is crucial to improve dentists’ knowledge, practice, and attitudes towards TD. Integration of TD topics into undergraduate/postgraduate curricula is highly recommended. Special attention should be directed to training general dental practitioners and junior dentists.

## 1. Introduction

Teledentistry (TD) has emerged as an important communication tool in dental practice [1]. It involves remote communication between dentists and patients using information technology means such as smartphones, Internet, video calls, and social media channels [1,2]. Hence, dental practitioners (DPs) can provide dental advice, make provisional diagnosis, and educate their patients over a distance [1,2,3]. TD has been applied in different disciplines of dentistry, including but not limited to, preventive dentistry, endodontics, orthodontics, and oral medicine settings, with promising outcomes [2,4,5,6]. A recent systematic review and meta-analysis of 19 studies found that TD was a very effective means for oral health improvement in terms of prevention and promotion [7]. Another systematic review of 10 studies indicated that TD can successfully be implemented in the diagnosis and detection of dental caries [8]. The key advantages of TD include cost-effectiveness, providing access to dental care in rural areas and for frail people, reducing wait times, and making referrals in an efficient way [5,6,7,8,9,10]. In addition, TD is an opportunity for peer education and consultation through video conferencing, facilitating better dental care for patients [2].

The unexpected rapid spread of COVID-19 has caused an unprecedented public health crisis and changed the landscape of the health system worldwide [1,2]. Consequently, the popularity of TD has increased during the COVID-19 pandemic [3]. In response to the exponential spread of the pandemic, with the aim of slowing down the spread of the disease, most countries imposed measures including social distancing and complete or partial lockdowns lasting several months. In line with this, most dental services in the majority of countries were suspended, and dental care was limited to emergency visits only [11,12]. The resultant delays in diagnosis and management of dental diseases led to devastating consequences in oral health conditions [13,14]. This prompted DPs to resort to the use of alternative means (mainly phones, WhatsApp, and video conferencing) in order to follow up with their patients, provide dental advice, and even prescribe medications to emergency cases [3]. Indeed, the COVID-19 pandemic is not the first, nor will it be the last, and unless we are prepared, the cost in terms of human life and economy will be unimaginable. This certainly emphasizes the importance of TD application in different fields of dentistry. However, it can be argued that the efficiency of TD application in dental practice is guaranteed only if the DPs have sufficient knowledge and training on how to practice TD. Unfortunately, despite its importance, recent surveys have shown that DPs have poor knowledge and lack adequate experience in TD [15,16]. In Saudi Arabia, perception of TD among DPs is not clear. Hence, the present study aims at assessing the knowledge, attitudes, and practice of TD among DPs in Saudi Arabia and the association with their qualifications and clinical experience.

## 2. Materials and Methods

### 2.1. Study Design and Participants

The current study was an online questionnaire-based survey targeting DPs in Saudi Arabia. The protocol of the study was reviewed and approved by the Institutional Review Board of the Scientific Research Unit at the Vision College of Dentistry and Nursing in Riyadh, Saudi Arabia (IRB No: alf.dent-2020032). The sample size was calculated using the OpenEpi statistical software (https://www.openepi.com/SampleSize/SSCohort.htm, accessed on 23 October 2021), considering 95% confidence interval, 80% power, and an expected 50% level of knowledge. The estimated sample size was 376 participants.

### 2.2. Survey Instrument

The survey tool was a self-administered questionnaire that was adapted from previously conducted studies [17,18]. The main survey was preceded by a pilot one among 20 DPs to assess the clarity and validity of the contents, and the questionnaire was modified accordingly. The final survey presented the objective of the study and invited participants to provide their consent. Participants were assured of the confidentiality and anonymity of the obtained data. The questionnaire comprised 26 items, divided into four parts. The first part of the questionnaire sought to collect the demographic information of the participants. The second part included seven questions to assess knowledge and practice regarding TD. The third section comprised nine questions regarding the attitudes of participants towards TD. A five-point Likert scale was used to measure DPs’ attitudes. The two extremes of the Likert scale were strongly agree and strongly disagree. The last part of the survey comprised 3 TD-training related questions.

### 2.3. Data Collection

Several WhatsApp, Twitter, and Instagram groups of practicing dentists in Saudi Arabia were identified by the authors. Simultaneously, an electronic form of the questionnaire was prepared on survey monkey (https://www.surveymonkey.com/welcome/sem/?program, accessed on 28 October 2021) and shared through the assigned social media platforms to a convenient random sample of DPs in Saudi Arabia (*n* = 1908). The purpose of the study was explained, and the targeted dentists were contacted in person and invited to participate. Data collection continued over a four-month period, and the survey was closed by May 2021.

### 2.4. Data Analysis

The SPSS statistical package was used for data analysis (IBM SPSS Statistics for Windows, Version 22.0, Released 2011, IBM Corp., Armonk, NY, USA). Descriptive statistics presented demographics of participants and frequency tables, and bar and pie charts presented responses of the participating dentists to the survey questions. The Chi-Square statistic was used to assess the association between questionnaire items and DPs’ qualification/clinical experience. A *p*-value < 0.05 was considered significant.

## 3. Results

A total of 603 participants (227 interns, 281 general dentists, 95 specialists) completed the questionnaire, giving a response rate of 31.6%. Around 50% of the participants were females, with a mean age of 28.8 ± 5.8 (range: 21–60 years). Out of the total, the majority (85%) were Saudis, 71% had been in practice for 5 years or less, and 46% came from the central region of Saudi Arabia. In addition, 34.5% worked in university clinics, 34% in the private sector, 28% in the public sector, and 3% practiced in both the private and public sectors. Table 1 presents the characteristics of the study population.

As presented in Table 2, the participants revealed poor knowledge of TD, with only 38% having heard about TD and 37.3% knowing what TD was, with significant differences depending on participants’ qualifications and years of clinical experience (*p* > 0.01). Generally, specialists showed significantly better knowledge compared to dentists and interns. Similarly, years of clinical experience was significantly associated with better knowledge (*p* > 0.01). Less than one-third (28.7%) of the participants thought that TD was applicable in all branches of dentistry, with no significant differences between the groups.

Concerning the current practice of TD, only one-quarter of the subjects (23.2%) reported currently practicing TD, with no significant differences based on qualification or years of experience (*p* < 0.05). Around 19.6% had started applying TD the previous year, while only 6% had started earlier (i.e., ≥2 years ago). When asked about their intention to use TD in the future, 66.3% showed their interest in using and applying TD in their daily practice, with no significant differences based on qualification or years of experience (*p* < 0.05) (Table 2).

**Figure 1:**
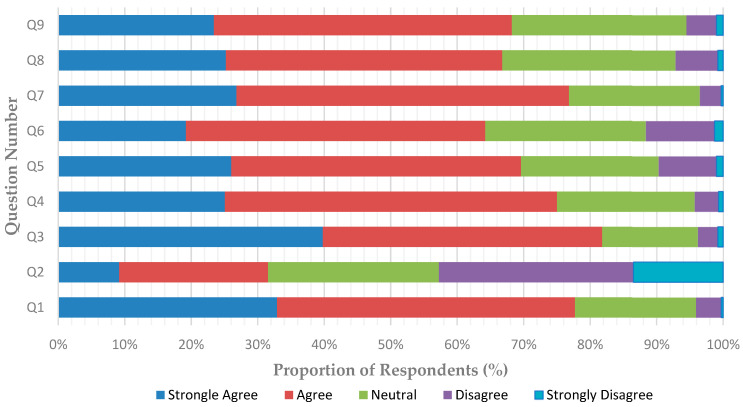
Responses of participants to questions related to attitudes toward teledentistry (*n* = 603).

The participants’ responses to questions on attitudes and opinions regarding TD are depicted in Table 3 and Figure 1. Their answers (strongly agree/agree) to questions on attitudes varied greatly, ranging from as low as 31.3% to as high as 81.4%. While the majority (81.4%) believed that TD is a good tool for oral hygiene instruction and that TD can improve the access to oral health services in rural areas (75.9%), only 31.3% of the participants agreed that dental examination using TD is as effective as traditional examination in the office setting. Almost 77% agreed that TD reduces patient flow during pandemics, and 66% thought that TD will play an important role in the future. Generally, although insignificant in most of the answered items, specialists and practitioners with greater experience showed more positive attitudes toward TD (Table 3).

**Figure 2:**
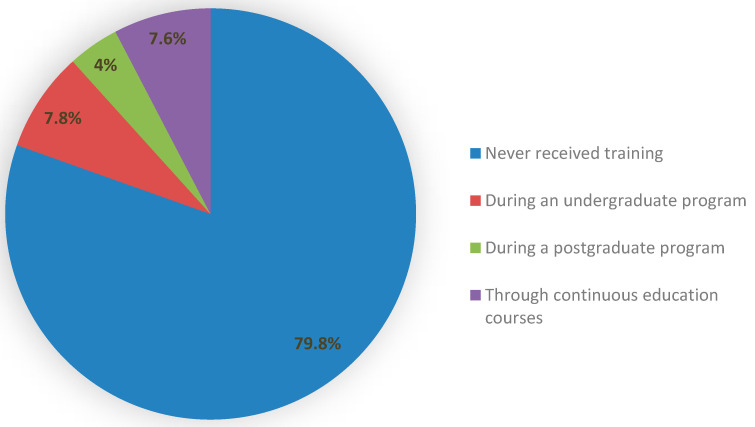
Participants’ responses regarding training received on teledentistry.

Only 20.2% of the participants reported having attended at least one workshop or a lecture about TD over the last year, with significant differences between the participants based on qualification (specialists 34.4% vs. general dental practitioners 18.3% and interns 17.3%; *p* = 0.001) and years of experience (*p* = 0.001). Likewise, the majority (79.8%) reported that they hadn’t received any training on TD, whereas only 19.4% reported having had some training either during their undergraduate program (7.8%) or postgraduate program (4%) or through continuous education courses (7.6%) (Figure 2), with significant differences between the subjects based on their qualification and years of experience (*p* < 0.01). Around two-thirds of the participants felt that they need further training on TD. The aforementioned results are illustrated in Table 4.

## 4. Discussion

The development of current dental curricula and planning continuous dental education programs require careful assessment of the needs of contemporary dental practice and requirements of the job market. Dental practitioners worldwide should be aware of such needs/requirements and be equipped with the required knowledge and skills to provide standard oral/dental care and fulfill the ongoing needs/demands of their community. In this context, the current study can be used as a guide for dental educators and planners of oral health services in Saudi Arabia. Although TD is not a new discipline [19], the emergence of the COVID-19 pandemic raised the attention of the dental community about its important role as a tool for the provision of a wide range of remote dental services, with no risk of infection [20].

The key finding in the present study is the poor knowledge and practice of TD among DPs in Saudi Arabia. These results are consistent with earlier international studies that reported inadequate knowledge of DPs on TD [15,16]. Such findings can be attributed to the inadequate didactic and practical training on the topic in the dental curricula and a lack of continuous education programs on TD. Therefore, integrating TD in the undergraduate and postgraduate curricula is of paramount importance in order to fill the knowledge gap. Additionally, periodic continuous education programs (such as lectures and hands-on workshops) targeting all DPs are highly recommended.

Another important finding is that most of the participants expressed positive attitudes and willingness to practice TD in the future. This is in line with the findings of similar previous surveys in Saudi Arabia that show an encouraging atmosphere to adopt TD by DPs in the kingdom [18,21]. Similar findings were also observed among dentists from Pakistan, Brazil, and Australia [15,16,22]. The emerging perceived need for TD among DPs during the COVID-19 health crises may have resulted in these positive attitudes of DPs.

It is noteworthy that only 31.3% of the dentists in this study agreed that dental examination via online video calls and intraoral cameras is as effective as in the traditional office setting. Although this suggests uncertainty among most of the DPs regarding the reliability of dental examination through TD, the available literature indicates controversy regarding the accuracy of the diagnosis provided by TD [8,23,24]. Further research will shed more light on the factors that maximize the reliability of examination via the tools of TD.

One key barrier to the application of TD in dental settings is the lack of training. Unfortunately, most of the participants (79.8%) in the current study reported a lack of training on the use and application of TD. This in turn may have had a negative impact on their knowledge, practice, and attitudes toward TD. Interestingly, the self-perceived need for training on the various aspects of TD was apparent among the study population, as is evident by their willingness to take on courses on TD. This finding may help designers of continuing professional development programs in Saudi Arabia.

A recent survey among Brazilian dentists, dental specialists, and dentists with greater than 10 years of clinical experience showed more preparedness to practice TD in comparison with general DPs and dentists with less clinical experience [16]. In Saudi Arabia, awareness of TD among dental professionals was associated with qualification and work experience [18]. In agreement with the above studies, the results of the present survey indicate that dental specialists and dentists of longer clinical experience appear to surpass general DPs, intern dentists, and junior dentists in terms of knowledge, attitudes, and practice of TD. This can be attributed to the greater previous exposure of specialists and long-experienced dentists to workshops and training on TD, as a higher proportion of the latter groups reported attending at least one workshop/lecture and received some sort of training on TD. This again emphasizes the importance of periodic continuous education on TD, targeting all DPs in Saudi Arabia. It can be argued that future integration of TD in daily clinical practice in Saudi Arabia requires targeting the whole range of DPs, with special attention towards general DPs and junior dentists.

Use of a convenience sampling method and the low response rate are limitations of this study. However, the fact that characteristics of the study population comprised a wide range of DPs in Saudi Arabia may add to the credibility and generalizability of the findings.

In this study, less than a third of the participants (28.7%) agreed that TD is applicable in all branches of dentistry. An important question for further research is what are the branches of dentistry that are more appropriate for the implementation of TD? This should be coupled with the development of related guidelines and a legal/ethical framework for the purpose of standard dental/oral care that respects patients’ privacy and confidentiality.

Utilizing TD in dental practice requires adequate provision of the necessary infrastructure, such as networking, appropriate hardware, intraoral cameras, and digital images. However, it is not yet clear to what extent Saudi dental hospitals/centers/clinics are prepared to practice TD. Future qualitative research may address preparedness, current barriers, and potential mechanisms for the wider integration of TD in the armament of oral health services in Saudi Arabia. The perspective of patients is also of paramount importance and merits investigation.

## 5. Conclusions

The results revealed poor knowledge, training, and practice of TD among DPs in Saudi Arabia. However, the positive attitude expressed by most of the participants towards practicing TD in the future is an encouraging sign for dental educators and planners of oral health care. Continuous education through periodic workshops and training courses on TD is crucial to improve DPs’ knowledge, practice, and attitudes towards TD. The integration of TD topics into undergraduate/postgraduate curricula is highly recommended. Special attention should be directed to training general DPs and junior dentists.

## Figures and Tables

**Table 1:** Characteristics of participants (*n* = 603).

**Age (years)** Mean (SD) 28.8 (5.8) Range 39 Minimum 21 Maximum 60	**Gender** Male 299 (49.6%) Female 304 (50.4%)	**Nationality** Saudi 510 (84.6%) Non-Saudi 93 (15.4%)
**Practice Location in Saudi Arabia ** Northern Region 45 (7.5%) Southern Region 76 (12.6%) Central Region 280 (46.4%) Eastern Region 132 (21.9%) Western Region 70 (11.6%)	**Type of Practice** Governmental 169 (28%) Private 206 (34.2%) Both 20 (3.3%) University Clinics 208 (34.5%)	**Clinical Experience (years)** Mean (SD) 4.9 (4.6) <=5 years 426 (70.6%) >5 years 177 (29.4%)
**Qualification** Dental Intern 227 (37.6%) DDS/BDS 281 (46.6%) Postgraduate Qualification 95 (15.8%)		

**Table 2:** Responses of participants to knowledge- and practice-related questions by qualification and clinical experience (*n* = 603).

Question	% of “Yes” Answers Based on Qualification and Clinical Experience							
	Total	Qualification			*p*-Value	Clinical Experience		*p*-Value
		Intern	GP	Specialist		≤5 Years	>5 Years	
Knowledge Items								
Have you ever heard about TD?	38	35.7	32.4	60	<0.001 *	33.8	48	0.005 *
Do you know what TD is?	37.3	36.1	32.7	53.7	0.001 *	34.7	43.5	0.128
“TD is the practice of using computers, Internet, and technologies for dental consultation and treatment planning over a distance”. Do you agree with this definition?	72.6	71.8	70.1	83	0.013 *	72.1	74.4	0.839
Do you think that TD is applicable in all branches of dentistry?	28.7	33	24.6	30.5	0.59	28.9	28.4	0.363
**Practice items**								
Do you practice TD at your current workplace?	23.2	18.2	26.4	26.6	0.069	22.2	26.1	0.302
When did you start practicing TD?								
*Never*	73.8	81.9	70.7	66.7	0.004 *	75.7	70.9	0.109
*1 year ago*	19.6	11	24	28		18.8	21.7	
*≥2 years ago*	6	7.1	5.4	5.4		5.5	7.4	
In the future, will you practice TD?	66.3	66.8	69.3	57.9	0.384	68.4	62.1	0.280

* Denotes significant difference at *p* < 0.05 as indicated by Chi-Square statistics.

**Table 3:** Responses of participants to attitude-related questions based on qualification and clinical experience (*n* = 603).

Question	% of “Strongly Agree/Agree” Answers Based on Qualification and Clinical Experience							
	Total	Qualification			*p*-Value	Clinical Experience		*p*-Value
		Intern	GP	Specialist		≤5 Years	>5 Years	
Q1. TD can reduce patient flow during pandemics by postponing non urgent dental visits.	77.4	78.3	77.1	77.9	0.028 *	76.9	79.5	0.143
Q2. Dental examination via online video calls and intraoral cameras is as effective as in the traditional office setting.	31.3	22	39.4	31.2	<0.001 *	29.9	35.4	0.035 *
Q3. TD is a good tool for giving oral hygiene instructions to patients.	81.4	80.1	82.5	84	0.540	80.4	85.2	0.100
Q4. TD can be an addition to the regular care that dentists provide.	74.8	75.3	72.5	82	0.822	75.3	74.4	0.307
Q5. TD saves time for the dentist.	68.7	66.1	72.5	69.6	0.211	69	70.9	0.006 *
Q6. TD can help in reducing costs for dental practices.	63.9	60.8	67.5	62.4	0.402	61.8	69.9	0.189
Q7. TD can improve the access to oral healthcare services, particularly in rural areas and during pandemics.	75.9	80.8	71.5	83	0.023 *	74.1	83.5	0.038 *
Q8. TD will have a major role in future clinical practice.	65.9	63.7	68.5	68.9	0.705	66.5	67.4	0.490
Q9. There is a clear need for a government initiative in the form of a TD program whereby patients could obtain advice on treatment needs from a central facility.	67.6	64.6	70.6	69.9	0.003 *	68	68.9	0.006 *

* Denotes significant difference at *p* < 0.05 as indicated by Chi-Square statistics.

**Table 4:** Responses of participants to training-related questions based on qualification and clinical experience (*n* = 603).

Question	% of “Yes” Answers Based on Qualification and Clinical Experience							
	Total	Qualification			*p*-Value	Clinical Experience		*p*-Value
		Intern	GP	Specialist		≤5 Years	>5 Years	
**Training items**								
Have you attended a workshop/lecture about TD in the last year?	20.2	17.3	18.3	34.4	0.001 *	16.7	29.3	0.001 *
Have you ever received training on the use and application of TD?	19.4	18.1	17.2	30.1	<0.001 *	17.3	25.2	0.006 *
Do you feel a need for training/further training on the use and application of TD?	69.7	72.6	70.6	62.1	0.276	72.6	63.8	0.064

* Denotes significant difference at *p* < 0.05 as indicated by Chi-Square statistics.

## Data Availability

The data that support the findings of this study are available on reasonable request from the corresponding author (M.Z.N). The data are not publicly available due to privacy or ethical restrictions.
